# Increasing pediatric radiation oncology capacity in sub-saharan Africa using technology: a pilot of a pediatric radiation oncology virtual training course

**DOI:** 10.1186/s12909-024-05313-5

**Published:** 2024-03-20

**Authors:** Adedayo O. Joseph, Adeseye M. Akinsete, Azeezat O. Ajose, Aishat T. Oladipo, Adeola Maliki, Korede Akindele, Michelle Mangongolo, Samuel Adeneye, Wilfred Ngwa

**Affiliations:** 1 NSIA – LUTH Cancer Centre, Lagos University Teaching Hospital, Lagos, Nigeria; 2 Hematology & Oncology Unit, Department of Pediatrics, College of Medicine, University of Lagos, Lagos, Nigeria; 3 The Dorcas Cancer Foundation, Lagos, Nigeria; 4 Johns Hopkins School of Medicine, Baltimore, MD USA

**Keywords:** Medical Education, Pediatric Radiation Oncology, Pediatric Cancer, Africa, Virtual training, Technology, Professional development, Radiotherapy, Capacity building

## Abstract

**Background:**

The shortage of skilled healthcare professionals in pediatric oncology and the limited access to training programs remain significant challenges in Nigeria and sub-Saharan Africa. The the Pediatric Radiation Oncology (Virtual) Course, ‘PedROC’ project aims to contribute to improving pediatric cancer outcomes in Nigeria by increasing the capacity of radiation oncology professionals. To address the gap in access to pediatric radiation oncology professional development, the PedROC project was created, harnessing technology to improve radiation oncology training via a curriculum delivered through web-conferencing. This study aimed to evaluate the effectiveness of the PedROC pilot in enhancing the capacity, confidence, and skill of radiation oncologists in decision-making, prescribing, and treatment planning of radiotherapy for children diagnosed with cancer.

**Methods:**

A multidisciplinary faculty of specialists in radiation oncology, pediatric oncology, oncology nursing, radiation therapy technology, and medical physics collaborated to identify the key learning needs in pediatric radiation oncology in the country. The team collaborated to develop a comprehensive curriculum covering the most common pediatric cancers in sub-Saharan Africa for the training program. The training course was conducted over two days, delivering twenty-four half-hour sessions for a total of 12 h, from July 31 to August 01, 2021.

**Results:**

Analysis of pre and post - training surveys showed a significant increase in self-reported confidence measures across all domains among radiation oncologists. The program successfully improved participants’ knowledge and confidence levels in managing common pediatric cancers using radiotherapy, particularly addressing radiotherapy-specific issues such as appropriate dose, target volume delineation, treatment planning, dose constraints, and plan evaluation.

**Conclusion:**

The PedROC pilot showed the efficacy of this model in enhancing the capacity and confidence of radiation oncology professionals involved in the treatment of pediatric cancer. The findings indicate that technology holds significant potential to increase pediatric radiation oncology capacity in Africa, ensuring improved access to proper treatment and ultimately improving pediatric cancer outcomes.

## Background

Ward et al. (2019) estimated 397,000 cases of pediatric cancer diagnosed globally in 2015, and projected there would be a total of 6.7 million new cases worldwide from 2015 to 2030 [1, 2]. GLOBOCAN data from 2018 estimate 43,649 annual cancer cases among children living in Africa [2]. More than 80% of pediatric cancer cases occur in low- and middle-income countries (LMIC), with an estimated 5-year survival rate of 37.4%, making cancer a leading global cause of pediatric disease burden [3, 4]. The 5-year survival rate of pediatric cancer in LMIC is significantly lower than in high-income countries [5].

Pediatric cancer in Africa is beleaguered by challenges such as limited awareness of pediatric cancer, limited access to quality healthcare services, delayed and, or missed diagnosis, insufficient funding, and shortage of expertise. There is limited specialized cancer care, expertise, and infrastructural resources in sub-Saharan Africa (SSA) further hindering the provision of treatment and contributing to consistently poorer outcomes for children with cancer [6, 7]. The disparity in access to pediatric cancer care in SSA is a contributing factor to lower survival rates ranging from 8.1 to 30.3%. compared to 80% recorded in high-income countries [6, 8].

Radiotherapy is an essential component of the multimodal treatment of pediatric cancer. Advanced techniques that deliver more conformal treatments with lower doses to organs at risk reduce the potential for long-term adverse effects in pediatric patients [5, 9]. Parkes et al. (2017) posit that the number of cancer cases requiring radiotherapy is expected to increase significantly by 2035, with over 7 million cases projected to occur in LMICs [9]. This is higher than projected numbers for high-income countries(HICs). It is further estimated that a large proportion of these new cases will be in the pediatric population, thus increasing demand for pediatric radiotherapy in LMICs [9]. 

Despite this rising demand for pediatric radiation, there persists a significant lack of access for children in low and middle income countries worldwide and especially in Africa. This lack of access is attributable to factors such as inadequate infrastructure, insufficient funding, and a shortage of skilled manpower [10]. The expected rise in demand for pediatric cancer treatment on the background of the current deficit in expertise necessitates investment in the pediatric radiation oncology workforce in Africa.

The Global Initiative for Childhood Cancer (GICC), launched in 2018 by the World Health Organization aims to double the estimated survival rate of pediatric cancer in LMICs to 60% by 2030 [3]. The deficiency of pediatric radiation oncologists and allied specialists, exacerbated by a limited availability of training programs threatens the potential achievement of this target [5, 11, 12]. A review of current literature revealed that there is currently little to no subspecialty training in pediatric radiotherapy available in Africa; for radiation oncologists, medical physicists, or therapy technologists [10, 13]. Taking into consideration regionally available resouces versus ongoing need, innovative approaches are necessary to increase and improve access to specialized pediatric radiation oncology training [12]. The use of digital technology and digital platforms for continued education of healthcare professionals has demonstrated outcomes comparable to traditional in-person teaching methods [14]. These technology-powered platforms eliminate time, distance and travel costs and constraints, in addition to reducing infection exposure of trainers and trainees [14].

According to the Lancet Oncology Commission on Sustainable Care for Children with Cancer, the development of competency-based curricula using distance learning, e-learning, and mobile digital education can rapidly and efficiently provide training to a large pool of healthcare professionals [12]. In a recent study assessing the effectiveness of online training for radiation oncologists, Hatcher et al. documented the effectiveness of a telehealth brachytherapy training program in increasing the confidence of healthcare professionals in LMICs [15]. Sandhu et al. (2022) also reported findings from a multi-institutional case-based radiation oncology virtual education rotation for residents; finding that the sessions significantly increased capacity to treat in majority of participants [16].

Harnessing the power of technology to connect professionals across geographic and time zones, a pediatric radiation oncology course, the Pediatric Radiation Oncology (Virtual) Course - PedROC, was created. This remote training program aims to deliver remote training sessions by leveraging expert pediatric radiation radiotherapy professionals worldwide. The goal is to enhance the capacity of practicing radiation oncology professionals in Africa, equipping them to effectively deliver radiation therapy to pediatric cancer patients. Using a web-conferencing platform, the curriculum was delivered via didactic sessions teaching on the epidemiology, clinical presentation, diagnosis, and management of the most common pediatric cancers; with a specific focus on radiotherapy delivery. This study evaluated the impact of the program pilot on the confidence levels of radiation oncology professionals practicing in Africa, supposing that increased knowledge and confidence levels would positively impact their abilty to deliver radiotherapy to pediatric cancer patients.

## Methods

### Development and implementation of the PedROC program

A multidisciplinary team comprising oncology professionals from West Africa and the United States, engaged in detailed discussions and collaborative decision-making to identify key learning needs for healthcare providers in pediatric cancer. The team utilized a combination of available needs assessment literature, clinical experience, and international guidelines (residency program curricula, IAEA residency training curriculum) to make a list of potential topics. Keeping in mind that it would require several sessions and editions to cover all of pediatric radiation oncology, the team arrived at a consensus on the essential topics to be covered in the Pediatric Radiation Oncology (Virtual) Course (PedROC) pilot that was feasible to deliver over two days. The resulting curriculum covered epidemiology, clinical presentation, diagnosis, and treatment of pediatric cancer with a specific emphasis on radiation oncology.

The training was delivered remotely via a web-conferencing platform through twenty-four (24) half-hour sessions delivered over 2 days from July 31, 2021, to August 1, 2021. The program was delivered in English and registration open to all healthcare professionals involved in pediatric cancer care, including radiation oncologists, medical physicists, radiation therapy technicians or therapy radiographers, radiologists, pediatric oncologists, pediatricians, nurses, residents, and fellows. Sessions were divided between basic disease presentation and principles of management that included surgical and chemotherapy treatment modalities, with focused attention to radiotherapy target and normal tissue delineation and radiation treatment planning and evaluation. Attendee questions were answered live during the sessions and email addresses of tutors were shared with attendees for follow-up questions not addressed during the live sessions.

The program was delivered in morning and afternoon sessions over two days. The morning sessions covered broad based topics such as: the burden of pediatric cancer in Nigeria and sub-Saharan Africa, imaging in pediatric oncology, building a pediatric tumor board, basic concepts in pediatric radiation, history of pediatric cancer treatment, differences between adult and pediatric oncology, basic concepts in pediatric radiation oncology and indications for radiation therapy in children. Afternoon sessions covered more specific topics such as nursing care of pediatric cancers, behavioral and play therapy techniques, pediatric sedation for treatment, and specifically radiotherapy treatment of common pediatric cancers such as nephroblastoma (Wilms’ tumor), pediatric central nervous system (CNS) tumors, bone and soft tissue sarcomas, and nasopharyngeal cancer. These sessions focused on dose determination, target delineation (contouring), treatment planning, and plan evaluation. Other topics included International Commission on Radiation Units and Measurements (ICRU) 50, 62, 71, and 78, normal tissue dose constraints, craniospinal irradiation contouring and planning, and radiotherapy for pediatric metastatic disease.

### Study and program design

This was a prospective interventional study, aimed to assess the effectiveness of technology as a tool to improve access to pediatric radiotherapy training among professionals practicing in Africa. The goal of the pilot program was to assess the impact of a remote training course on the confidence level of practicing radiation oncologists in Africa by assessing self-reported confidence levels before and after the course via digital pre- and post- training questionnaires.

### Faculty

The program had a diverse group of experts as faculty, representing a range of specialties including radiation oncology, pediatric hematology-oncology, radiology, and clinical research. The faculty comprised thirteen speakers in total, nine of whom were from outside Africa and four from within Africa. The faculty from outside Africa included a Radiation Oncologist with the Princess Margaret Cancer Centre in Canada, an Assistant Professor of Radiation Oncology and a fourth-year Radiation Oncology resident physician both at the University of Florida, a renowned retired Pediatric Hematologist-Oncologist who had worked for years with the St. Jude Children’s Research Hospital Tennessee, an Associate Professor of Radiation Oncology at the University of Pennsylvania, a Radiation Oncologist at the Massachusetts General Hospital in Boston, an Associate Professor of Radiation oncology at Harvard Medical School, and an Assistant Professor in the Department of Radiation Oncology at the University of Toronto. The trainers from within Africa included a Radiation Oncologist at the Korle Bu Teaching Hospital in Ghana, a Radiologist at Korle Bu Teaching Hospital in Ghana, a Professor of Radiotherapy and Oncology at Ahmadu Bello University Teaching Hospital, Zaria Nigeria, and a Professor of Pediatric Hematology-Oncology, and a consultant Pediatric Hemato-Oncologist at the University of Lagos College of Medicine, Nigeria.

### Participants

Participants for the PedROC pilot were recruited through a targeted approach, with efforts to include a diverse representation of healthcare professionals involved in pediatric cancer care. To achieve this representation, invitations were extended through emails to professional networks and associations of radiation and pediatric oncology professionals within Nigeria and Africa. An open registration link was shared via social media on LinkedIn, Instagram, and X (formerly known as Twitter). The official announcement for the program listed the target audience as radiation oncologists, medical physicists, radiation therapy technicians, radiologists, pediatric oncologists, pediatricians, and nurses involved in pediatric cancer care.

**Fig. 1:**
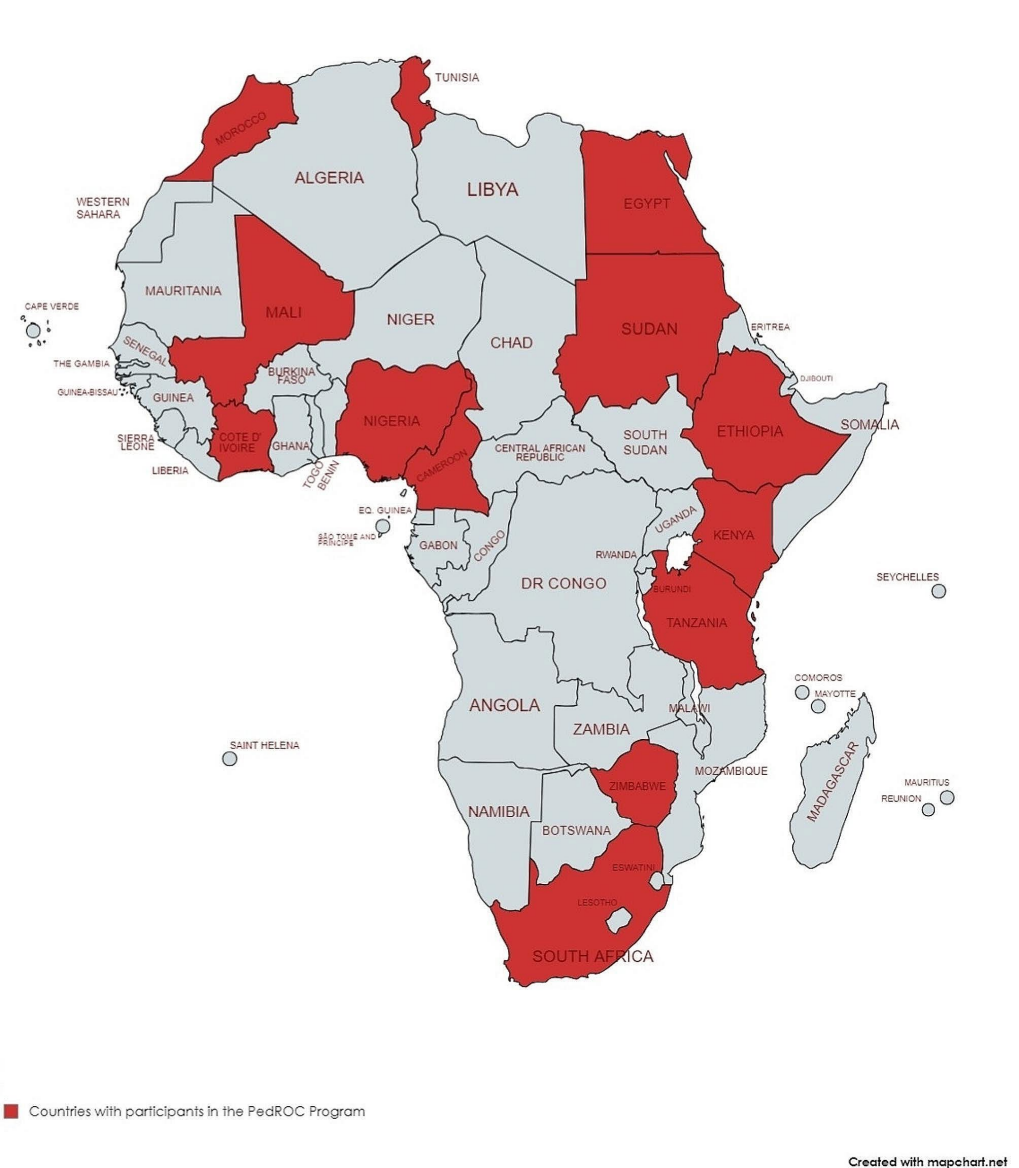
Map of Africa Showing Countries with Participants in the PEDROC program

The pilot aimed to have 50 attendees at each session, and attendance was recorded for both the participating centers and individual attendees. Attendees who attended at least 70% of the sessions received a certificate of completion. The program received a total of 276 registrations from 15 countries, 14 countries in Africa and 1 in South America, far exceeding the expected number. Of the 276 people who registered, 257 professionals, including 84 Radiation Oncologists, 46 Pediatric Oncologists, 23 Pediatricians, 47 Medical Physicists and Radiation Therapists, 17 Radiologists, 24 Nurses, and 16 other health workers representing 14 African countries attended the training sessions. (Fig. 1).

### Evaluation

We evaluated the pilot by measuring the self-reported impact of the program on confidence levels related to pediatric radiation treatment. Surveys were distributed via email to all attendees before (pre-training survey) and after the program (post-training survey). Consent was obtained from all participants before filling out the online survey form. Whilst the program received a diverse group of attendees, for the purpose of this pilot study, only responses from radiation oncologists were analysed. The survey assessed participant demographics, prior radiation oncology training, self-reported confidence levels on the topics covered in the training, and challenges to treating pediatric cancer in their country and institution. Attendees rated their confidence level using a 5-point Likert scale. The post-training survey included additional informal questions on session satisfaction, as well as feedback to direct future training.

### Statistical analysis

The data collected from the attendees were analyzed using SPSS Statistics version 26. The presentation of the data was in the form of frequencies with percentages (for participant demographic and satisfaction variables) and means with standard deviations (for knowledge and confidence variables). The study compared pre- and post-session self-reported confidence levels for each topic, as well as the average of all topics. Results were considered statistically significant when the *p*-value was less than 0.05.

## Results

257 healthcare professionals, including 84 radiation oncologists, 46 pediatric oncologists, 23 pediatricians, 47 medical physicists and radiation therapists, 17 radiologists, 24 nurses, and 16 other health workers from 14 African countries attended the sessions (Fig. 1).

Of the 84 radiation oncologists who attended the program, 66 responded to the pre-training survey and 40 to the post-training survey, with a total of 38 attendees who responded to both included in this analysis. Among the 38 radiation oncologists who were included in this analysis, 13 (34.2%) were consultants while 25 (65.8%) were resident doctors still in training. Most of the attendees were from Nigeria (14, 36.8%), followed by Ethiopia (7, 18.4%). Others were from Kenya, Ghana, Morocco, South Africa, Sudan, Morocco, Tanzania and Colombia. Half of the attendees (19, 50.0%) were within the age range of 25–35 years. The majority of the attendees reported that they had no previous specialized training in pediatric radiation oncology (24, 63.2%). Of those who had received training in pediatric radiation (14, 36.8%), the length of prior pediatric radiation training ranged from 1 week to 9 years. (Table 1)

**Table 1:** Socio-demographic characteristics of Radiation Oncologists (*n* = 38)

Variable	Frequency (Percentage)
**Age Range**	
25–35 years	19 (50.0%)
35–45 years	17 (44.7%)
> 45 years	2 (5.3%)
**Cadre**	
Consultant	13 (34.2%)
Resident Doctor	25 (65.8%)
**Country**	
Nigeria	14 (36.8%)
Ethiopia	7 (18.4%)
Kenya	5 (13.2%)
Ghana	3 (7.9%)
Tanzania	2 (5.3%)
South Africa	1 (2.6%)
Morocco	1 (2.6%)
Others	5 (13.2%)
**Work Experience**	
< 2 years	22 (57.9%)
2–5 years	13 (34.2%)
> 5 years	3 (7.9%)
**Previous Training in Pediatric Radiation Oncology**	
Yes	14 (36.8%)
No	24 (63.2%)
**Length of previous training (** ***n*** ** = 14)**	
< 1 month	4 (28.6%)
1–12 months	6 (42.9%)
> 1 year	4 (28.6%)

Age range and length of work experience did not significantly influence mean confidence level. Professional cadre was found to have a significant influence, with consultants reporting higher confidence levels compared to residents. Previous training in pediatric radiation oncology was found to be a significant factor, with those who had received training reporting higher confidence scores than those who had not. (Table 2). Self-reported confidence measures increased significantly on comparing post-training to pre-training evaluation responses across all domains. (Table 3).

**Table 2:** Association of Demographic Factors with Self Rated Confidence level among radiation oncologists before the program (*n* = 38)

VARIABLE	Mean (Standard Deviation)	df	*p*
**Age Range**			
25–35 years	1.90 (0.70)	2	0.548
35–45 years	2.02 (0.62)		
> 45 years	2.20 (0.70)		
**Cadre**			
Consultant	2.43 (0.49)	1	**0.04**
Resident Doctor	1.80 (0.64)		
**Work Experience**			
< 2 years	2.00 (0.64)	2	0.065
2–5 years	1.86 (0.65)		
> 5 years	2.83 (0.15)		
**Previous Training in Pediatric Radiation Oncology**			
Yes	2.69 (0.28)	1	**< 0.001**
No	1.63 (0.47)		

**Table 3:** Self-reported knowledge and confidence among radiation oncologists (*n* = 38)

Variable	Pre-Test*	Post-Test*	t	*p*-value
Knowledge of pediatric cancers	2 (0.735)	2.92 (0.882)	-5.050	**< 0.001**
Knowledge of indications for pediatric radiation treatment	2.08 (0.784)	2.97 (0.636)	-6.634	**< 0.001**
Knowledge of tolerance doses of pediatric organs at risk	2.26 (0.724)	3.32 (0.471)	-8.849	**< 0.001**
Knowledge of ICRU 50,62,71 and 78	2.08 (0.85)	3.24 (0.59)	-7.791	**< 0.001**
Knowledge of staging pediatric malignancies	2.11 (0.798)	3.34 (0.627)	-7.255	**< 0.001**
Confidence in radiation treatment planning for pediatric CNS tumors.	2.03 (0.716)	3.11 (0.689)	-7.544	**< 0.001**
Confidence in radiation treatment planning for Wilms tumor.	2 (0.805)	3.16 (0.638)	-7.133	**< 0.001**
Confidence in radiation treatment planning for pediatric sarcomas.	1.95 (0.837)	2.97 (0.545)	-6.164	**< 0.001**
Confidence in contouring pediatric normal structures.	1.95 (0.733)	3.26 (0.601)	-8.976	**< 0.001**
Confidence in contouring pediatric target volume structures on CT/MRI imaging.	1.74 (0.724)	3.13 (0.665)	-8.830	**< 0.001**

a. Confidence scores were rated on a 5-point Likert score

b. Pre-and post-test scores are mean (standard deviation)

## Discussion

There is a shortage of pediatric radiation expertise and limited professional development opportunities globally. This shortage is even more pronounced in SSA, despite rising pediatric cancer incidence [17]. This deficit is a barrier to care for children with cancer in sub-Saharan Africa, and a potential contributor to consistently sub-optimal pediatric cancer treatment and lower survival rates. To address this, innovative approaches to increase pediatric radiation expertise in Africa are necessary, leveraging the rise of technology and partnerships with international organizations and experts. Remote continuing medical education utilizing technology is valuable in overcoming the limits of regional resources; and eliminating geographic, distance, time, and travel variations and restrictions [18]. Virtual training programs present an opportunity to improve radiation oncology capacity without extensive or expensive travel [14, 18]. This approach can also help standardize training across the continent, ensuring consistent high-quality curriculum instruction and delivery for pediatric radiation oncology across Africa.

The PedROC program aims to increase and improve pediatric radiotherapy expertise through remote delivery of didactic lectures from an expert faculty with pediatric oncology and pediatric radiation experience and track record. The survey findings from the program pilot indicate that technology can be effectively employed to improve self-rated confidence and potentially capacity in pediatric radiation among radiation oncologists practicing in Africa. The positive impact of a remote training program as observed in our study, is consistent with data from other studies. Hatcher et al. (2020) reported the efficacy of an online brachytherapy training course among health workers in 10 countries with reported confidence scores improving significantly in all competencies evaluated [15]. As early as 2012, Alfieri et al. had reported a significant improvement in mean test scores among Canadian radiation oncology residents after a web-based interactive radiologic anatomy and treatment planning training module [19].

The pre-training survey also revealed baseline gaps in the training and confidence levels of radiation oncologists with regards to pediatric radiation oncology. Our findings are similar to that of Kavuma et al. who also reported low confidence levels in using IMRT among radiation therapy professionals in low-and-middle income countries, including Africa [20]. The majority of surveyed radiation oncologists had not received any previous pediatric radiotherapy-specific training despite working in centers that provide care to pediatric cancer patients. The baseline confidence levels of the participants varied across different aspects of pediatric radiation oncology; the highest baseline confidence levels were observed in the knowledge of staging pediatric cancers. This is similar to findings by El Khababi et al. where the use of a dedicated virtual training course significantly increased diagnostic confidence and staging confidence among radiologists [21]. This could be attributed to the fact that staging is a fundamental aspect of cancer management and is emphasized in general oncology training. Despite this higher baseline confidence, there was room for improvement in specific areas related to pediatric cancer staging. The lowest baseline confidence levels were in pediatric target volume delineation on CT and MR images (contouring), and in treatment planning for pediatric sarcoma. This finding suggests that radiation oncologists in Africa would benefit from additional training and support in specific areas of pediatric radiation planning and delivery. Addressing this gap through targeted training programs could improve the quality of care provided to children diagnosed with cancer on the continent.

The highest change in confidence levels identified after the training was in contouring target volumes on CT and MR images. This indicates that the program had a significant positive impact on participants’ skill and confidence in this critical aspect of pediatric radiotherapy. Kavuma et al. (2023) reported similar results after providing training to 37 healthcare professionals from Uganda, Guatemala, and Mongolia; finding that the remote training significantly improved the average experience and confidence level in contouring, site-specific target/organ definition, planning/optimization, and quality assurance amongst radiation oncologists, medical physicists, radiation therapy technologists and dosimetrists [20]. Accurate delineation of target volumes is absolutely essential for precise radiation treatment, and the observed improvement in confidence levels could be extrapolated to assume improved capacity in this skill [22].

The PedROC program is an educational intervention dedicated specifically to enhancing access to pediatric radiation expertise in Africa using technology; thus making it the first of its kind on the continent. 257 participants, including 84 radiation oncologists from 14 African countries, participated in the pilot, taught by a faculty of 14 regional and international experts. The online approach facilitated a broad representation of participants and faculty in terms of geographic distribution and professional backgrounds. It provided access to faculty from diverse cancer centers across three continents and several time zones, a feat that would be financially and logistically difficult to accomplish physically.

The pilot program was attended by professionals across the radiation oncology continuum, including medical physicists, radiologists, medical oncologists, pediatric oncologists, nurses, and radiation therapy technologists with some breakout sessions specific for physicists, therpists, and nurses. As each of these specialties have different educational and expertise needs and it would be ideal to tailor evaluations to the specific requirements of each specialty in order to adequately assess the effectiveness of the program. The large sample distribution with diverse specific professional duties made it challenging to evaluate changes in confidence for all specialties and the pilot study focused on evaluating the impact of the training program on radiation oncologists only. Pre- and post-course surveys assessing confidence levels in prescribing, contouring, and evaluating treatment plans were administered to radiation oncologists. Non - clinical general feedback and participant satisfaction surveys were administered to other groups of participants such as medical physicists and radiation therapy technologists following the course. This feedback was valuable in gauging the immediate impressions and experiences of these participants, the potential impact of the course on their practice, and suggestions for ensuring and improving the value of the course.

We acknowledge the limitation of not having specifically assessed all groups of radiation professionals such as physicists and therapists in our study. While the curriculum was designed to cover all aspects of radiotherapy contouring, planning, and treatment delivery, it was deemed important to work systematically through the different specialties, especially for the pilot program. Future iterations would work to develop strategies to assess the program’s impact on all specialties involved in pediatric radiotherapy decision making, treatment planning and treament delivery. This will involve the development of survey instruments tailored to the unique roles and responsibilities of each healthcare discipline, ensuring a more extensive and nuanced examination of the program’s impact on various participants’ confidence levels and skills.

Other limitations were a relatively small sample size, intensified by non-response from some participants despite efforts to encourage survey participation; potentially limiting the generalizability of the findings. This should however be examined in the context of the relatively low number of radiation oncologists and even lower pediatric radiation centers or professionals in the region. Further, the convenience sampling method composed primarily of participants who voluntarily enrolled in the course may not fully represent the diversity of healthcare professionals involved in pediatric cancer care in Africa.

Finally, the online delivery format, while increasing convenience and overcoming distance and availability barriers, may pose its own unique barrier to uptake. Some participants reported technological challenges such as audio or visual malfunctions, which may have hindered their completion of certain modules. To overcome this limitation, alternative methods such as distribution of on-demand videos need to be explored. Future studies may also consider incorporating control groups to more deeply evaluate the program’s comparative effectiveness. Ongoing evaluation and feedback from participants would be used to continuously refine and update the program content and delivery methods.

## Conclusion

This report of the Pediatric Radiation Oncology (Virtual) Course (PedROC) pilot demonstrates the effectiveness of a remote medical educational intervention to improve the confidence and capacity of radiation oncology professionals in the treatment of pediatric cancers. The program had expected to enrol a maximum of 50 participants for the pilot. The attendance of 257 particpants from 14 African countries emphasizes the existence of a training gap and need in pediatric radiation oncology in Africa. It is important for the African continent to prepare to adequately respond to the growing population of pediatric cancer patients in coming years. By prioritizing the sustainability of this program, we can foster continuous professional development and enhance the quality of care for children with cancer in Africa. Future work from the PedROC program is directed at expanding the course to hone in on specific cancers per edition, delivering curricula specific to the represented disciplines in radiation oncology, creating intra- and inter- country and continent networking opportunities, and facilitating mentoring relationships.

## Data Availability

The datasets used and/or analyzed during the current study are available from the corresponding author upon reasonable request.

## Abbreviations

PedROC: Pediatric Radiation Oncology (Virtual) Course
GLOBOCAN: Global Cancer Observatory
LMIC: Low- and Middle-Income Countries
SSA: sub-Saharan Africa
HIC: High-income countries
GICC: Global Initiative for Childhood Cancers
ICRU: International Commission on Radiation Units and Measurements

## References

[1] Ward ZJ, Yeh JM, Bhakta N, Frazier AL, Atun R (2019). Estimating the total incidence of global childhood cancer: a simulation-based analysis. Lancet Oncol.

[2] Sharma R (2021). A Systematic Examination of Burden of Childhood Cancers in 183 countries: estimates from GLOBOCAN 2018. Eur J Cancer Care.

[3] Graetz DE, Garza M, Rodriguez-Galindo C, Mack JW (2020). Pediatric cancer communication in low- and middle-income countries: a scoping review. Cancer.

[4] Magrath I, Steliarova-Foucher E, Epelman S, Ribeiro RC, Harif M, Li C-K (2013). Paediatric cancer in low-income and middle-income countries. Lancet Oncol.

[5] Hernandez S, Nguyen C, Parkes J, Burger H, Rhee DJ, Netherton T (2023). Automating the treatment planning process for 3D-conformal pediatric craniospinal irradiation therapy. Pediatr Blood Cancer.

[6] van Heerden J, Zaghloul M, Neven A, de Rojas T, Geel J, Patte C, et al. Pediatric Oncology clinical trials and Collaborative Research in Africa: current Landscape and Future perspectives. JCO Global Oncol. 2020;1264–75. 10.1200/GO.20.00159 10.1200/GO.20.00159 PMC7456323 32762563

[7] Geel JA, Challinor J, Ranasinghe N, Myezo KH, Eyal KC, Aderounmu W (2021). Pediatric cancer care in Africa: SIOP Global Mapping Program report on economic and population indicators. Pediatr Blood Cancer.

[8] Zandaki D, Sultan I, Al-Shamsi HO, Abu-Gheida IH, Iqbal F, Al-Awadhi A (2022). Pediatric Oncology in the Arab World. Cancer in the Arab World.

[9] Parkes J, Hess C, Burger H, Anacak Y, Ahern V, Howard SC (2017). Recommendations for the treatment of children with radiotherapy in low- and middle-income countries (LMIC): a position paper from the Pediatric Radiation Oncology Society (PROS-LMIC) and Pediatric Oncology in developing countries (PODC) working groups of the International Society of Pediatric Oncology (SIOP). Pediatr Blood Cancer.

[10] Hess CB, Parkes J, Janssens GO, Lin C, Wong K, Zaghloul MS (2021). Global pediatric radiation therapy in resource-limited settings. Pediatr Blood Cancer.

[11] Khader J, Al-Mousa A, Al Khatib S, Wadi-Ramahi S (2020). Successful development of a competency-based Residency Training Program in Radiation Oncology: our 15-Year experience from within a developing country. J Canc Educ.

[12] Atun R, Bhakta N, Denburg A, Frazier AL, Friedrich P, Gupta S (2020). Sustainable care for children with cancer: a Lancet Oncology Commission. Lancet Oncol.

[13] Kortmann R-D, Freeman C, Marcus K, Claude L, Dieckmann K, Halperin E (2016). Paediatric radiation oncology in the care of childhood cancer: a position paper by the International Paediatric Radiation Oncology Society (PROS). Radiother Oncol.

[14] Doherty M, Rayala S, Evans E, Rowe J, Rapelli V, Palat G. Using virtual learning to Build Pediatric Palliative Care Capacity in South Asia: experiences of implementing a Teleteaching and Mentorship Program (Project ECHO). JCO Global Oncol. 2021;210(22). 10.1200/GO.20.00481 10.1200/GO.20.00481 PMC8081544 33555911

[15] Hatcher JB, Oladeru O, Chang B, Malhotra S, Mcleod M, Shulman A, et al. Impact of high-dose-rate Brachytherapy Training via Telehealth in Low- and Middle-Income Countries. JCO Global Oncol. 2020;1803–12. 10.1200/GO.20.00302 10.1200/GO.20.00302 PMC7713515 33216647

[16] Sandhu NK, Rahimy E, Hutten R, Shukla U, Rajkumar-Calkins A, Miller JA et al. Radiation Oncology virtual education rotation (ROVER) 2.0 for residents: implementation and outcomes. J Cancer Educ 2022:1–8. 10.1007/s13187-022-02216-1 10.1007/s13187-022-02216-1 PMC9461407 36083458

[17] Paulino AC, Dieckmann K, Esiashvili N, Mahajan A, Janssens GO, Halperin EC (2020). Training and education of pediatric radiation oncologists: a survey from the 2019 Pediatric Radiation Oncology Society meeting. Pediatr Blood Cancer.

[18] Balogun O, Rodin D, Ngwa W, Grover S, Longo J (2017). Challenges and prospects for providing Radiation Oncology services in Africa. Semin Radiat Oncol.

[19] Alfieri J, Portelance L, Souhami L, Steinert Y, McLeod P, Gallant F (2012). Development and impact evaluation of an E-Learning Radiation Oncology Module. Int J Radiation Oncology*Biology*Physics.

[20] Kavuma A, Kibudde S, Schmidt M, Zhao T, Gay H, Li B (2023). Remote global Radiation Oncology Education and Training: a pathway to increase Access to High-Quality Radiation Therapy services in low- and Middle-Income Countries. Adv Radiation Oncol.

[21] El Khababi N, Beets-Tan RGH, Tissier R, Lahaye MJ, Maas M, Curvo-Semedo L (2024). Outcomes and potential impact of a virtual hands-on training program on MRI staging confidence and performance in rectal cancer. Eur Radiol.

[22] Lin R (2014). Target volume delineation and margins in the management of lung cancers in the era of image guided radiation therapy. J Med Radiat Sci.

